# Bibliographical Notices

**Published:** 1845-07

**Authors:** 


### Pulmonary Consumption, successfully treated with Naphtha, &c. Second Edition. By *John Hastings*, M.D., Senior Physician to the Blenheim Street Dispensary. Octavo, pp. 260. London, 1845. Churchill.

Our notice of the first edition of this work, in the number of this Journal for October 1843, will be found to contain the pith and kernel of its contents. Those, who choose to give a trial to the Naphtha, must take care that it is of the proper quality. The right sort is, chemically speaking, *pyro-acetic spirit*, obtained by the distinctive distillation of an acetate, generally of lead or lime: the dose is from ten to fifteen drops and upwards, mixed with a table-spoonful of water, three times a day. Hydriodate of potash, digitalis, colchicum, and other remedies, that may be deemed advisable, can be given along with it.

Dr. Hastings assures us that his enlarged experience of the remedy has tended to increase his confidence in its remedial powers. Would to heaven that other medical men may be conscientiously able to confirm the accuracy of his opinion! That our author is a hasty man in his conclusions is abundantly apparent from the circumstance that he regards Belladonna to be quite as decidedly a specific for scarlet fever as Vaccination is for the prevention of small-pox. He introduces the reports of several cases which have appeared in the pages of the weekly periodicals since the publication of the first edition of his work, and in which benefit seemed to be derived from the use of the Naphtha. In one of these, we notice a curious discrepancy of opinion as to the diagnosis of the seat of the pulmonary lesion between two eminent hospital physicians, both authors and acknowledged stethoscopists. The first wrote as follows:—"I think there is obstruction in the *left* lung, most probably crude tubercles." The other, who examined the patient three days afterwards, wrote:—"I find collapse and dulness below the *right* clavicle, with decidedly tubular breath-sound and voice. There certainly are tubercles at the apex of the *right* lung."

Dr. Hastings, at the close of his work, narrates the particulars of the two cases in which Mr. Storks has recently made, under his direction, an opening through the thoracic parietes into a tubercular cavity of the lungs. One of the patients, operated upon last November, is still alive; and his health, although still very precarious, appears to be better than it was then. In the other case, the patient died 14 days after the operation; which, in our opinion, was quite unjustifiable. Dissection revealed two large irregular cavities, occupying about two-thirds of the right (it was on this side that the opening had been made) lung, and a small one in the summit of the left one! The only circumstances, in which we should think any medical man warranted in making an opening into a purulent cavity in the lungs, would be where there was the apparent pointing of an abscess outwardly over the seat of the supposed vomica—an occasional, although certainly a rare, occurrence. This was the case in two most interesting observations, which occurred several years ago in the practice of Sir Henry Marsh of Dublin. Our readers will doubtless read with interest the following account of one of these, as briefly related by Sir Henry himself. "The patient, a young woman, 23 or 24 years of age, was admitted into Steeven's Hospital, exhibiting the usual signs, local and constitutional, of tuberculous phthisis; the former being limited, apparently, to the superior part of the right lung. In that situation there existed unequivocal signs of a tubercular abscess. After having remained for some time in hospital, there was observed, about two inches above the right nipple, a small, red, slightly elevated spot upon the skin, which appeared like the usual pointing of an abscess. At this point a free incision was made between the ribs, and through the opening a large quantity of tubercular matter, pus, and thin transparent fluid escaped. This remained for months a fistulous opening, giving exit to matter in varied quantities; during the whole of that time, I remember that a candle, placed close to the opening, was extinguished by a forcible expiration. There was scarcely a day for many weeks that a candle was not so placed by some one or other of the pupils, and always extinguished during expiration or coughing. The girl improved greatly in health, from the hour of the performance of the operation. The hectic fever, the night sweats, the emaciation, the cough and expectoration, all ceased; the chest contracted, and the strongest expectations of ultimate recovery were entertained; we were, however, disappointed: rather suddenly, all the formidable symptoms re-appeared, and the auricular signs of tubercles in the left lung were no longer doubtful. She died, manifesting all the characters of acute phthisis; the fistulous opening discharging to the last. All I can remember of the dissection is, that in the right lung there were presented the thickened irregular walls of a large tubercular abscess, nearly empty; the disease being absolutely limited to the superior third of this lung, whilst, in the left lung, the appearances were those of a recent and abundant deposition of tubercles."

In the other case—which occurred in a young girl who had hectic fever, cough, and purulent sputa—the examination of the chest detected no signs of disease, except under the left clavicle; there well-marked *gargouillement* existed. "After a few days the abscess began to point externally, a lancet was introduced, and much apparently purulent matter escaped: the chest fell in gradually, the quantity of matter discharged diminished daily; and the constitutional signs of pulmonary disease subsided. A year afterwards I saw this little girl, well nourished, free from fever, cough, and expectoration, and restored to excellent health. The chest on the left side remained much contracted; it was, in the infra-clavicular region, comparatively dull on percussion, and the respiratory sounds in that situation were comparatively feeble but pure; in all other parts of the thorax the auricular signs were normal."

---

An Essay on Marriage; being a Microscopic Investigation into its Physiological and Physical Relations, with Observations on the Nature, Causes and Treatment of Spermatorrhea, &c. By *R. Dawson,* M.D. &c. 8vo. pp. 102. London, 1845. Hughes.

The title-page of this work did not certainly prepossess us much in its favour, as we immediately began to think of Messrs. Goss and Co. Dr. Courtenay, and other men of that stamp, whose names are to be seen in almost every newspaper that we take into our hands. Our first business therefore was to look at the author's name and qualifications, and we found that he writes himself, "Licentiate of the Royal College of Physicians, London, M.R.C.S.E. &c." Notwithstanding, however, these vouchers of his respectable education, Dr. Dawson has in our opinion acted with very questionable propriety in affixing his name to such a production as this.

essay; bearing, as it does, all the characters of a book, alike by its name and contents, that is less calculated for the enlightenment of his professional brethren, than for attracting the notice of the public, or at least of such members of it as wish to know something of what Dr. Ryan called the Philosophy of Marriage! Be this as it may, we can have no hesitation in assuring our readers that Dr. Dawson's Essay contains nothing that will repay the trouble of perusal.

It may, indeed, be well to remind them that, in long-continued and obstinate gleets, there may be a certain degree of seminal weakness or Spermatorrhœa present, and that the only way to determine this point of diagnosis conclusively, is to examine the urethral discharge with the microscope. If any seminal fluid be present, the existence of Spermatozoa may always be thus detected.

As a matter of course, in the treatment of such cases, we should trust rather to the use of cold bathing, steel, &c. than to that of internal or external astringents. Dr. Dawson recommends Lallemand's practice of gently cauterising the urethra with the nitrate of silver, introduced by means of his "porte-caustique."

Minute doses of corrosive sublimate with sarsaparilla are of great service, whenever the epididymis, or the testicle generally, is hardened and irregular. To check the filthy practice of the onanist, it will be useful to irritate the surface of the penis with the tartar-emetic ointment, or the acetum lyttæ; keeping up the irritation, if we can, for a month or six weeks at a time, and thereby preventing, all the while, the continuance of self-pollution.

---

THE PHILOSOPHY OF FEMALE HEALTH; being an Enquiry into its connection with, and dependence upon, the due Performance of the Uterine Functions, &c. By *S. Mason*, M.D. Licentiate of the Royal College of Physicians, London, and M.R.C.S.E. &c. 8vo. p. 107. London, 1845. Hughes.

OUR lurking suspicions of the character of the work, which we have just noticed, are amply confirmed by the perusal of the present one. It would seem that Drs. Dawson and Mason live together, and carry on, we presume, a joint concern. The one manages all the gentlemen patients, curing them of Impotence and suchlike delicate infirmities; while the other takes the ladies under his care; his attention being, it would seem, chiefly directed to the interesting subject of Sterility, and the means of obviating this sad bar to conjugal happiness. The two partners may have agreed, upon due consideration, that it would give some eclat to their establishment, if they both appeared in print at the same time. Hence most probably the origin of their twin literary productions.

Dr. Mason's "Philosophy of Female Health" is unquestionably very much worse than Dr. Dawson's "Essay on Marriage," being little better than the general run of works of professed empirics. The only thing in it worthy of any notice is, that he says that he has found an etherial extract of Chio turpentine—especially when combined with the extract of Cubebs—in doses of from five to ten grains twice or thrice a day, exceedingly useful in the disorders usually connected with atony or irregular action of the uterus. Steel medicines may often be given at the same time with much benefit.

---

HINTS TO MOTHERS AND OTHER PERSONS INTERESTED IN THE MANAGEMENT OF FEMALES AT THE AGE OF PUBERTY. By *Jonathan Toogood,* Licentiate of the Royal College of Physicians, F.R.C.S.E., and Senior Medical Officer of the Bridgewater Infirmary. 8vo. p. 20. London, 1845. Churchill.

THESE "Hints" are surely not worthy of the respectable name which they bear. That the health of young females at the age of puberty, and the first setting in of the catamenial secretion, requires to be narrowly watched, is, as a matter of course, perfectly well known to every enlightened medical man. Dr. Toogood more than once alludes to the great advantage of "a plan which will be detailed hereafter," in the treatment of Chlorosis, Amenorrhœa, and the various maladies so often connected with these states; but, strange to say, he has omitted to inform us of what the plan consists: all that we are told being simply "that aperients and tonics—particularly some of the preparations of steel—are the remedies from which relief is chiefly to be obtained." There is certainly nothing very novel in this.

We regret to observe that he thinks it worth while to give one or two extracts from the letters of "a lady of high rank and great intelligence, who had witnessed the beneficial effects of judicious treatment in similar cases, and who applied to me, in behalf of this patient;"—very interesting truly! In another page we read that one of Dr. Toogood's patients, who had been cured by his plan, "afterwards induced several of her friends who resided in Ireland to consult me, by representing the benefit she had derived from the treatment adopted." Surely such scraps of information are not what the profession has a right to expect from so respectable a member of their corps as Dr. Toogood.

---

ON THE REMEDIAL INFLUENCE OF OXYGEN, NITROUS OXYDE, AND OTHER GASES, ELECTRICITY AND GALVANISM, &c. By *J. E. Riadore,* M.D. F.L.S. &c. 8vo. pp. 177. London, 1845. Churchill.

THE general character and tone of this work may be judged of by giving one or two short extracts. The reader will doubtless admire the logical accuracy of Dr. Riadore in grouping together the following very analogous diseases:—

"A general scepticism prevails as to the curing of tubercular phthisis, scrofula, asthma, chronic bronchial affections, paralysis, nervousness, and some forms of cutaneous affections; in truth, there are no diseases which bid such general defiance to the means of medical art. Some members of the medical profession, however, doubt even the possibility of success in some of these cases; but to them and all I can most confidently declare that all my cases are related with the strictest fidelity, and that I have in every instance studied rather to under-rate the results than the contrary."

The shadowy and comprehensive generality of the expression, "some forms of cutaneous affections," will not fail to attract attention.

By turning to page 43, we find an account of Dr. Riadore's practice in various diseases.

"I have found inhalation of oxygen of very great use in cases of indigestion, nervous debilitated conditions of the liver and kidneys, asthma, paralysis, uterine affections; and, externally applied, it is of benefit in chronic swellings of the joints."

In the course of his remarks on Electricity, we meet with the following curious piece of information, "I have frequently been informed by my patients that they have passed dead worms after I had galvanised them for some other affection. This fact may be somewhat analogous to the well known effect of a thunder-storm upon insects, leeches, worms, fishes, &c."

The bulk of the work is taken up with loose rambling remarks on food, various gases, electricity, galvanism, &c. eked out with numerous quotations from the writings of various authors, more especially of Liebig. There is an ugly Chapter on "Disorders of the generative organs," which much reminds us of the contemporaneous productions of Drs. Dawson and Mason. Do let us entreat of Dr. R. to pause before carrying into effect his announced intention to "publish his experience, in a distinct treatise, on spermatorrhœa or involuntary emission, and other defective functions of the generative organs." We are also promised, ere long, a report of our author's experience on the effects of electro-galvanism for the destruction of urinary calculi!

---

### PRACTICAL NOTES ON INSANITY. By *John Burdett Steward*, M.D. F.R.C. of Physicians, &c. 12mo. pp. 122. London, 1845. Churchill.

IN this little work, the author has described the results of his experience as *physician to the Droitwich Lunatic Asylum for the period of ten years.* There is no novelty in it, nor anything which calls for particular notice from us. Dr. Steward appears to be a sensible intelligent man, not given to speculation, nor ambitious of distinction by the peculiarity of his views and opinions. Perhaps the following remarks on the use of Emetics will enable the reader to judge for himself of the general quiet tone of these "Practical Notes."

"Properly used, emetics are of the first importance in symptomatic mania; and, fortunately, their administration is easy, without having recourse to the objectionable custom of giving them in food. Where the general health is good, and where no other cause for treatment is apparent, than mere noise or violence, the use of so powerful and painful a remedy is seldom justifiable. It is not because a patient is out of temper, or because he may be a little more extravagant than usual, that we are justified in giving an emetic; on the contrary, we ought to witness the existence of symptoms pointing out the propriety of its exhibition, independent of maniacal affection; we ought to have a flushed countenance, a rapid pulse, a high temperature, a furred tongue, and cold extremities; or at least we ought to suspect constipation, or an over-loaded stomach. To sum up our duty in a few words, we are bound to entertain a definite and sufficient object, in the use of this, or any other remedy, before we employ it."

---

### Some Observations on Organic Alterations of the Heart, and particularly on the beneficial Employment of Iron in the Treatment of such Cases. By *S. Scott Alison*, M.D. &c. 12mo. pp. 62. London, 1845. Longman and Co.

The object of this little work is to recommend the more general exhibition of Steel medicines, and consequently to avoid the use of all lowering means, in various organic diseases of the heart, which are unaccompanied with any symptoms of inflammation or sanguineous plethora. That chalybeates are often useful in those cases of cardiac disease, in which we have reason to believe that there is either an atony in the muscular parietes of the heart, or an impoverished condition of the blood—provided always the secretion of the liver and other chylopoietic viscera be duly attended to—has very generally been admitted by all the best writers on this branch of pathology. Dr. Elliotson strongly recommended them in his clinical lectures at St. Thomas's Hospital, a good many years ago; and Dr. Copland also alludes in a forcible manner to the benefit to be derived from their use. Still however, many medical practitioners are too apt to have recourse to antiphlogistic and lowering remedies, more especially of digitalis, in almost all cases of supposed hypertrophe of the heart; seeming to forget that there may be a positive deficiency of proper muscular power, with an inordinate thickness of its muscular parietes. Dr. Alison's remarks are very well calculated to remove this error, and to lead the reader to adopt a more rational and successful treatment. We have repeatedly had occasion to observe the good effects of a regulated course of chalybeate medicines—none is better than the common sesqui-oxyde, taken in any bitter infusion: Dr. A. seems to prefer the Mist. ferri comp., and the Ferri iodidum—in cases of *atonic* disease of the heart. Much of the benefit is unquestionably owing to their invigorating action upon the stomach, the comfort of the patient always depending very materially upon the state of his digestive organs. Hence the great and often immediate benefit that is obtained, in such cases, from the use of alkalis, bitters, hydrocyanic acid, and mild opiates. We have already—vide the review of the works of Drs. Latham and Furnival in the present number of this Journal—spoken of the paramount importance of a seton or issue in the cardiac region, in almost all cases of hypertrophic enlargement. Dr. Alison makes no mention of the remedy.

---

### A few Remarks on Hydropathy, &c. By *William F. Soltau*, M. B. è Coll. Ball. Oxon. Octavo, pp. 72. London and Plymouth. 1845.

This *brochure* ought surely to have made its appearance a year or two ago; as the cold water cure has been evidently on the wane for some time past—witness the frequent advertisements of late for the sale of (once flourishing) Hydropathic Establishments. We need scarcely say that Dr. Soltau, like every other sensible man, ridicules and condemns this and all such attempts to impose on the credulity of the public, to the sacrifice of their health as well as of their substance.

How comes it that our Oxonian author has allowed a very obvious error to creep into the very first sentence of his work? The well-known aphorism of Hippocrates is surely "Ο βιος βραχὺς, ἡ δὲ τέχνη μακρὴ"—not μαχρή.

---

### The Lost Senses: Deafness. By *John Kitto*, D.D. 12mo, pp. 206. London, 1845. Knight.

A little volume that will well repay perusal. It is written by the editor of the Pictorial Bible, and contains a truly interesting account of his deafness, which is complete and absolute, and is the result of an injury of the head when he was only 12 years of age. The heaviest clap of thunder is perfectly inaudible by him; and indeed no sound, however loud, unless it induces a vibration perceptible by the general sense of touch, makes any impression upon him. The effect produced by a piece of ordnance, fired off near him, is compared to the sensation of a heavy blow upon the head from a fist covered with a boxing-glove: it is *tactual* rather than *acoustic*. So sensitive, however, is the body to the impressions arising from the gentlest tangible percussions, that, when any small article, as a thimble, pencil, or even a more minute object, falls from the table upon the floor, Dr. K. is often aware of it, even when other persons sitting at the same table have not been apprised of it by the ear. He adds,

> "I am subject to a painful infliction from the same cause, during the hour in which my little ones are admitted to the run of my study. It often happens that the smallest of them, in making their way behind my chair, strike their heads against it: and the concussion is, to my sensation, so severe, that I invariably wheel hastily round in great trepidation, expecting to see the little creature seriously injured by the blow; and am as often relieved and delighted to see it moving merrily on, as if nothing in the world had happened."

We could gladly extend our extracts from this interesting and beautifully written work; but this will not be necessary, if our readers take our advice of reading it for themselves.

---

### Observations upon the Employment of Compression in Aneurism. By *O'B. Bellingham*, M.D., F.R.C.S.I. Octavo, pp. 14. Dublin, 1845.

Since the re-introduction of compression in the treatment of popliteal and femoral aneurism by Dr. Hutton, in 1842, there have occurred eleven other cases in which it has been successfully employed. Dr. Bellingham, under whose care two of these were, has, in two numbers of the Dublin Journal, detailed the progress of this vast improvement in surgery. Compression, as now exercised, differs from that heretofore essayed in its lesser degree and shorter duration. The object now is not to altogether obstruct the flow of blood through the artery, but only to diminish it: "a partial current through the sac enables the fibrine to be readily entangled in the parietes of the sac in the first instance, and this goes on increasing, until it becomes filled; the collateral branches having been previously enlarged, the circulation is readily carried on through them." Instead of one compressing instrument, two or three are employed if necessary, so that when the pressure of one becomes painful it may be relaxed, another having been previously tightened. The patient can even regulate this himself, and no suspension of treatment in consequence of the irritation produced is necessary. The Dublin surgeons employ an instrument invented by a patient, and consisting of a modification of a carpenter's clamp, and which, Dr. Bellingham believes causes far less pain than that employed in the London hospitals.

The advantages of compression over the ligature are—1. That it is not attended with the slightest risk. 2. It is applicable to certain cases to which the ligature is not, as well as to some in which the employment of the ligature might be attended with unfavourable circumstances. Thus in very large aneurisms, the pressure so long continued acts injuriously upon the collateral circulation, and the application of the ligature may be followed by gangrene of the limb. So, too, in large aneurisms, inflammation and suppuration of the sac very commonly follow the ligature. The vessel, again, between the tumour and the heart, may be in too diseased a state to admit of the safe application of the ligature. "Pressure is applicable to cases of the aneurismal diathesis, and when more than one aneurism exists at the same time; cases in which the operation by ligature is likewise contra-indicated; as well as to cases of spontaneous aneurism, occurring in individuals of intemperate habits, or of broken-down constitution, in which the surgeon with great reluctance would perform any operation. A few cases have been related, in which the operation by ligature failed in consequence of some irregular distribution of the artery above the aneurism." 3. If pressure fail to cure the aneurism, it does not preclude, but favours, the successful performance of the operation.

The objections which have been offered to this plan are so frivolous—so purely, in some instances, objections for objection's sake, that we have neither patience or space for allusion to them. Hunter's procedure was a vast improvement upon the methods of his predecessors; but all must rejoice at the probability of even his painful and sometimes dangerous operation being speedily superseded by a new bloodless achievement in surgery.

---

### OBSERVATIONS ON THE MECHANISM AND DIAGNOSTIC VALUE OF THE FRICTION VIBRATIONS PERCEIVED BY THE EAR, AND BY THE TOUCH, IN PERITONITIS. By *Robert Spittal*, M.D., F.R.S.E. Pp. 19. [Reprinted from London and Edinburgh Monthly Medical Journal, May 1845.]

M. PIORRY states that although Laennec has not alluded to the subject in his work, he directed the attention of those who attended his *clinique* to the fact that the peritoneal surfaces, when inflamed, give rise to the production of certain audible sounds. M. Désprez, in 1834, stated to the Anatomical Society of Paris, that, in the early stages of peritonitis, a leather creaking or friction sound, analogous to that of pericarditis, could be heard. Since that period, Drs. Beatty, Bright, Stokes, Corrigan, and M. Désprez, have communicated additional observations: and Dr. Spittal details the particulars of two other cases in the present communication.

A tabular view is presented of the results observed in 15 cases, as recorded by the various authors. In the majority of these cases there was either a tumour of the abdomen present, or the peritoneal surface of one of the solid organs, as the liver or spleen, was affected; but the vibrations existed in other cases where these conditions did not prevail. Of the 15 cases, 4 recovered and the particulars of one are not stated. In 7 of the remaining cases, adhesions were discovered; but the *post-mortems* were in some instances made years or months after the first perception of the sounds, whose presence cannot be considered as diagnostic of the existence of adhesions.

"From the sixth column of the table it appears that almost every author who has made original observations upon this subject, has recorded the peculiar *vibratory indications perceived by the sense of touch over the part affected;* and this, no doubt, has arisen from the necessity of manual examination of the abdomen leading so directly to their detection. The character of the sensations has been very differently described, even in the same case, at different periods of the inflammation; so much so, indeed, as to render it more than probable that the various physical conditions of the serous surfaces had given rise to corresponding modifications of this peculiar sensation. * * * *The sounds perceived either along with the tactile vibrations*, or *unaccompanied* by them, are by no means so numerous as could have been wished for. Few as they are, however, they exhibit, as their identity of origin would have led us to expect, the same variations in character as the tactile vibrations, and so exact is the resemblance, and so much does the one indication suggest the other, that, in most instances, the same terms have been employed to describe them. * * * * * * * From the terms used to describe the vibrations perceived by the touch, it appears, that these sensations varied in intensity from a *soft creeping*, or *gentle vibration under the hand; or a sensation like that of the finger rubbed over a damp pane of glass*, to those of a more intense kind, described by the terms *creaking, crepitus*, and *grating*. The accompanying sounds varied in the same manner from a *gentle rustling*, to a *hard friction*, and sound of *creaking*."

The rubbing together of the peritoneal surfaces physically altered by the inflammatory process is the immediate cause of the vibrations. They are produced in three ways: by the respiratory movements, especially the descent of the diaphragm; by pressure with the hand; and by the peristaltic action. It is highly probable that in the early stages of peritonitis, when the membrane is drier than natural, friction vibrations occur as well as after the effusion of lymph. When the surfaces are separated by fluid effusion the vibrations cease, unless this be made to gravitate to some other part of the cavity than that under examination.

The author thus estimates the value of this sign.

"Of the value of the facts connected with the friction vibrations, it may be remarked, in conclusion, that they appear to have placed within our power an additional method of detecting the existence of an important and frequently fatal disease, often obscure by the ordinary modes of investigation, whether from peculiarity of idiosyncrasy, or from the co-existence of cerebral complication, by which pain and its characteristic results, with reference to the position of the patient, and to the respiratory movements, may have ceased to guide the physician in his diagnosis. * * * In cases of peritoneal inflammation in the upper portions of the abdomen, simulating pleuritis, the presence of any degree of the peristaltic friction vibration might very much assist us in the diagnosis."

---

### A Pentaglot Dictionary of the Terms employed in Anatomy, Physiology, Pathology, &c. &c. &c. In Two Parts.—Part Second and last. By *Shirley Palmer*, M.D., of Tamworth and Birmingham. Octavo, pp. 656. Longman and Co. 1845.

In our number for April, 1842, we spoke in high but just terms, of the first part of this gigantic—not in size but labour—production of Dr. Palmer. From some unaccounted cause, its appearance has been delayed four years after its completion. It is at length published, and will stand a memorial of its author's erudition and labour as long as any of the languages in which it is written. The love of fame, present and posthumous, must be very strong in the author of this Dictionary, who, amid the distractions of extensive private practice, must have devoted so much of his time and talents to its construction as scarcely to leave him a scanty opportunity for food and sleep during many years past! The "*auri sacra fames*" could not have entered into Dr. Palmer's calculation or ambition, since *profit* was totally out of the question. The constantly extending curricula of medical education, and, what is more, the daily increasing thirst for knowledge, render such a work as this indispensible to the student of our own days.

" The importance of an accurate knowledge of the French and German languages to the student of Medicine, few, in these enlightened times, will be ignorant enough to doubt, or have the effrontery to deny. Most auspiciously for the honour of our Profession and the interests of the public, such knowledge is, at length, recognized in the Schools, as an essential branch of medical education. Every attempt, therefore, to facilitate the attainment of these languages, and impart an additional impulse to the youthful and aspiring mind, even though it fail to acquire celebrity, must deserve encouragement and command respect.

" Of the profoundly scientific character and high practical value of the publications upon Medicine which are continually emanating from the continental press, none but they who have access to those productions of the Master-Spirits of our art in their original language, can form an adequate conception or correct estimate."—*Preface.*

Dr. Palmer informs us that, amidst the toils and anxieties of a provincial practice, he acquired, in his earlier years, without an instructor, a knowledge of the elements of several of the continental languages. The difficulties, which he experienced in this achievement, first suggested the idea of facilitating the labours of others by the extensive compilation now offered them at a comparatively trifling expense.

This devotion of our author to the good of his junior brethren is beyond all praise, and forms a remarkable exception to the host of modern authors who " rush into print," too often, for their own advantage, rather than that of the public! It would be useless to exhibit a specimen of this work; but we can safely recommend it to the attention of all zealous students as a treasure of inestimable value in the prosecution of their researches into the medical literature of the Continent.

---

### ON THE MEDICINAL PROPERTIES OF BEBEERINE. By *Douglas Maclagan.* M.D., F.R.S.E. (From the Ed. Med. and Surg. Journ. 163). Pp. 29. 1845.

BEBEERINE is a new vegetable alkali discovered by Dr. Rodie to exist in the Bebeera or Greenheart tree, a Lauraceous plant of British Guiana. From facts detailed in former publications, and in the present paper, Dr. Maclagan regards it as a powerful anti-periodic and tonic: and the cases he narrates bear him out in his estimate of its powers. As it can even now be produced at half the price of quinine, it will be rendered available in many instances in which that expensive drug is not so; and moreover, it has the great advantage of not injuriously affecting the nervous system to the same extent as quinine. Dr. Melier (See Medico-Chir. Rev. vol. 39, p. 306), has shewn that the enormous doses of quinine given in France have frequently a more or less distinctly poisonous effect; but even when given in quantities only sufficient to check a fully-formed intermittent, it occasionally, as observed by the author's correspondent, Dr. Watt of Demerara, produces very unpleasant symptoms. " The head becomes confused and feels larger than usual, with ringing in the ears and deafness. The whole nervous system appears to become affected, and sometimes the hands are so unsteady that the patient can scarcely write." Upon this point, however, conclusions must not be too hastily drawn, as in 5 out of

26 cases in which Bebeerine was employed, *tinnitus aurium* also occurred. At present, about 40 cases of intermittent or remittent *fever*, occurring in various climates, and at different ages, have been treated by Bebeerine; and in all of these it has manifested a more or less anti-periodic action, although in six it did not prove eventually satisfactory.

Professor Simpson of Edinburgh has given the drug an extensive trial in some other affections. He observes that Piorry and others represent quinine as sometimes inducing abortion, when given for the *periodic neuralgiæ of pregnancy;* and that he has used the Bebeerine with the most perfect success, even in some very severe cases of this kind. So, too, in cases of slight periodic fever, during puerperal convalescencc, he has found it succeed even after quinine, &c. had failed.

A case is also related of obstinate and repeatedly recurring *neuralgia faciei*, relieved from time to time by large doses of quinine, which, however, always produced "ringing of the ears, derangement of the digestive organs, and a severe febrile state of the system." In 1844, the sulphate of bebeerine was substituted, when the symptoms became effectually relieved, without any attendant inconveniences. The author thus concludes his paper:

"I formerly expressed my opinion that bebeerine differs from quinine in not being so liable to excite the circulation, or affect the nervous system; and this seems to be borne out by the above reports, especially those of the neuralgic cases; and it has been found useful by others as well as by myself in other cases where excitant action would be hurtful, as in cases of phthisis accompanied by atonic dyspepsia. With regard to the mode of administration of bebeerine, I have commonly given it in pill with conserve, in the same way and the same doses as quinine. It can also be readily given in the liquid form, the addition of a few drops of diluted sulphuric acid sufficing to form with it a perfect solution."

---

### Portrait of Sir William Burnett, K.C.H., M.D., F.R.S., &c., Director-General of the Medical Department of the Navy, &c. From a Painting presented by the Medical Officers of the Royal Navy. Price £2. 2*s*. and £1. 1*s*. Highley, 1845.

The original of this splendid engraving is, and ought to be, embalmed in the memory of every naval physician and surgeon, as one of the greatest benefactors of that important class of meretorious officers. Next to Lord Melville, the naval medical profession is more indebted to Sir William Burnett than to any medical commissioner since the American war. Neither Sir Gilbert Blane, Dr. Harness, nor Dr. Weir had the power, the energy, the perseverance to carry out the *details* of improvement, laying aside certain valuable *principles*, which the zealous Director-General of the present day has brought into effect by unwearied industry and untiring zeal. We hope soon to be able to give a sketch of this talented individual's labours; but in the mean time we strongly recommend all those who have not subscription copies, to supply themselves with one of the most faithful and admirable engravings we ever recollect to have seen.

### MEDICAL REFORM.

### REMARKS ON PHYSICIANS, SURGEONS, DRUGGISTS AND QUACKS. By Surgeon *Snipe*. 8vo. pp. 65. Highley, 1845.

OUR period of going to press will prevent our hearing Sir James Graham's "more last words" upon the subject of "Medical Reform;" and we must confess to entertaining some fears whether the advantages promised to the General Practitioner by the modified Bill (No. 3) may not, when even apparently within his grasp, either by his lukewarmness, the misguided conduct of some of his own body, or the opposition of his adversaries, even yet be wrested from him. The present Bill truly is not all that he requires or deserves, but it is certainly as much as in the present state of affairs he can reasonably hope to obtain, and is far more than he could have expected to have been able to wring out of the hostile clauses of the former measures. Sir James Graham has devoted time and attention to this subject, for which the profession cannot be too grateful, and all his later alterations have been favourable to the case of the General Practitioner. That he has approached the limit of these, beyond which he cannot venture, the newly-raised opposition of the Colleges shew; and we feel certain if he is by this opposition, or by the mistaken policy of the General Practitioners, persuaded to abandon or even postpone this Bill, the cause of Medical Reform will be indefinitely delayed. We look upon the opposition of the Council of the College of Surgeons—(that short-sighted body now aroused to the dangers of its suicidal policy without even yet perceiving the only honorable and effectual mode of amending it)—as the strongest of testimonials to the probable efficiency and respectability of the projected establishment. A mere modification of the Apothecaries' Society would have suited the Council very well; but the erection of an institution "co-ordinate in professional rank and importance with the existing Colleges of Physicians and Surgeons," is quite another thing. Really the impertinence of these gentlemen would be amusing, were it not for its practical consequences. They degrade the mass of their members by invidious and unjust preferences, they resist their ardent demands for an adjustment of differences, they insult them with documents, the perusal of which has excited one general storm of indignation; and yet, when these outcasts endeavour to contrive means for establishing their own future status and respectability, they step in and employ all their efforts to prevent their doing so! Bad as the conduct of the Council has been, we look upon any attempt at obliging them at once to amend it, as futile and delusive; and that a worse policy could not be followed than that of delaying the acceptance of the new Charter of Incorporation with this view. This Government will aid in no attempt at coercion, and none can be successful without their sanction. The views of the minority in the Council have never been explicitly stated, and are by no means satisfactory as far as they have been stated. We do not mean to say that the members are quietly to sit down under the injuries they have received; but the agitation for the removal of their grievances to be of avail must be of long continuance, and will operate doubtless eventually through the new electoral body: but to postpone for this remote and possible contingency, the reception of the benefits which are now offered to the profession would be sheer folly. If the present Bill can be postponed, the Council will have achieved a real triumph; whereas, if it be carried, they will be found far more disposed to effect some arrangement with their members, whereby their present position and influence may be in some measure retained if possible.

No great question, agitating such diverse parties was ever settled without some degree of compromise, and we look upon the present Bill to be as favourable a measure as the General Practitioner can at present procure—a tolerable foundation to commence with, but which will require and receive amendments hereafter. The penal clauses, although a considerable advance upon those contained in the Apothecaries' Act, will be found too clumsy and expensive for effectual working, and will we doubt not, after a while, be replaced by a summary jurisdiction. The preliminary examination, although it inflicts no kind of degradation upon the general practitioner, is a needless piece of complexity, no more required here than in Scotland or Ireland. It is a "one-portal" crotchet of Sir James, which, as the Council of the College themselves even strongly object to it, will probably soon be thrown overboard.

Surgeon Snipe is a droll fellow; but he much over-estimates the present condition of the public mind, when he believes that it suffices to exhibit the education and acquirements of the regular practitioner to insure his preference to the charlatan and druggist.

